# Germline-dependent transmission of male reproductive traits induced by an endocrine disruptor, di-2-ethylhexyl phthalate, in future generations

**DOI:** 10.1038/s41598-020-62584-w

**Published:** 2020-03-31

**Authors:** Radwa Barakat, Po-Ching Lin, Chan Jin Park, Mohamed Zeineldin, Sherry Zhou, Saniya Rattan, Emily Brehm, Jodi A. Flaws, CheMyong J. Ko

**Affiliations:** 1 Department of Comparative Biosciences, College of Veterinary Medicine, University of Illinois at Urbana-Champaign, Urbana, IL 61802 USA; 2 Department of Toxicology and Forensic Medicine, College of Veterinary Medicine, Benha University, Qalyubia, Benha, 13518 Egypt; 3 Carl R. Woese Institute for Genomic Biology, University of Illinois at Urbana-Champaign, Urbana, IL 61801 USA

**Keywords:** Endocrine reproductive disorders, Endocrine reproductive disorders

## Abstract

In males, defective reproductive traits induced by an exposure to an endocrine disruptor are transmitted to future generations via epigenetic modification of the germ cells. Interestingly, the impacted future generations display a wide range of heterogeneity in their reproductive traits. In this study, the role that the Y chromosome plays in creating such heterogeneity is explored by testing the hypothesis that the Y chromosome serves as a carrier of the exposure impact to future generations. This hypothesis implies that a male who has a Y chromosome that is from a male that was exposed to an endocrine disruptor will display a more severe reproductive phenotype than a male whose Y chromosome is from an unexposed male. To test this hypothesis, we used a mouse model in which F1 generation animals were exposed prenatally to an endocrine disruptor, di-2-ethylhexyl phthalate (DEHP), and the severity of impacted reproductive traits was compared between the F3 generation males that were descendants of F1 males (paternal lineage) and those from F1 females (maternal lineage). Pregnant dams (F0 generation) were exposed to the vehicle or 20 or 200 μg/kg/day of DEHP from gestation day 11 until birth. Paternal lineage F3 DEHP males exhibited decreased fertility, testicular steroidogenic capacity, and spermatogenesis that were more severely impaired than those of maternal lineage males. Indeed, testicular transcriptome analysis found that a number of Y chromosomal genes had altered expression patterns in the paternal lineage males. This transgenerational difference in the DEHP impact can be attributed specifically to the Y chromosome.

## Introduction

Epidemiological data consistently show a clear trend of decreasing semen sperm count and quality in men in the last few decades^1–3^. The cause of the declining sperm quality is not fully understood, but exposure to synthetic chemicals in the environment is regarded as a contributing factor^4–6^. Among them, plasticizers in consumer products are concerning because they are ubiquitous, in direct contact with humans, and known to disrupt the endocrine system^7,8^. As of 2015, the yearly global production of plastics reached 381 million tons, which is equivalent to the mass of two-thirds of the world’s population^8–11^.

Phthalates are synthetic plasticizers that are used primarily to improve flexibility and softness of polyvinyl chloride (PVC) plastic products^12^. One of the most widely used phthalates is di-2-ethylhexyl phthalate (DEHP), which is considered to be one of the most widespread environmental contaminants worldwide, with a production volume of 4 million tons per year^13–15^. DEHP is used in a broad range of consumer products such as food and beverage containers, insecticides, personal care products, medical equipment such as intravenous blood bags, packaging, children’s toys, and building materials^16,17^. DEHP is not covalently bound to the PVC polymer, and it easily leaches out into the environment and comes into contact with humans and animals through ingestion, inhalation, or dermal absorption^18,19^. Urinary DEHP metabolite concentrations indicate that human exposure to DEHP ranges between 3–30 µg/kg/day^20,21^.

As an endocrine-disrupting chemical (EDC), DEHP disrupts the reproductive system and acts as an anti-androgen in both females and males^22–24^. DEHP metabolites have been detected in amniotic fluid^19^, umbilical cord blood^25^, and other bodily fluids^26^, indicating that humans are exposed to DEHP as early as fetal stage of their development^27^. Indeed, exposure to DEHP during the fetal period increases the chances of epigenetic changes that have long-lasting developmental and functional impacts^28^. For instance, prenatal exposure to DEHP has been implicated in decreased anogenital distance, reduced testosterone levels, and poor semen quality^29–32^, and it accelerates reproductive aging, resulting in premature reproductive senescence in male as well as female mice^33,34^.

When a pregnant female (F0) is exposed to an EDC, the F1 generation is exposed as a developing pup, whereas the second (F2) generation is exposed as the developing germ cells in the gonad of the F1 male or female^35,36^. This means that the third (F3) generation is the generation not directly exposed to the EDC^36^. Interestingly, prenatal exposure to DEHP is reported to impact fertility and reproduction of F3 generation^37^. Previous studies showed that prenatal exposure to DEHP disrupts testicular germ cell organization and spermatogonial stem cell function in F3 generation^32,38^. An important mechanism for transgenerational transmission of early-life EDC exposure is thought to involve epigenomic reprogramming during development^36,39,40^. Therefore, EDC exposure that introduces epigenetic changes during early development permanently alters the epigenome in the germ line, and these changes can be transmitted to subsequent generations^41–43^. In contrast, when an EDC introduces epigenetic changes during adult life, the changes occur in somatic cells and are not transmitted to subsequent generations^44,45^. The transgenerational impacts are probably carried from one generation to the next via epigenetic modification^35,36,38,46^. Epigenetic inheritance can be modulated by environmental factors and transmitted to subsequent generations via germline cells^47^.

In a previous study, we reported that prenatal exposure to DEHP caused adverse effects in F1 males^33^. Specifically, we showed that prenatal exposure to DEHP accelerates reproductive aging and induces premature reproductive senescence, with an impairment of testosterone production and decline in sperm quality in the F1 male mice, but only after they were at least one year old. We followed the F1 generation males up to 22 months of age, as no obvious phenotype was seen at younger ages. Therefore, in this study, we kept the F3 males for more than one year so that we could follow their reproductive function at similar time-points as assessed in the F1 generation. Further, a number of studies have assessed the transgenerational impact of prenatal DEHP exposure on reproductive function and reported that DEHP induces reproductive dysfunction in the F2 and F3 generations^34,36,48,49^. Interestingly, the impacted future generations display a wide range of heterogeneity in their reproductive traits. In the current study, the role that the Y chromosome plays in creating such heterogeneity is explored by testing the hypothesis that the Y chromosome serves as a carrier of the exposure impact to future generations. This hypothesis implies that a male with a Y chromosome that is from a male that was exposed to an endocrine disruptor will display a more severe reproductive phenotype than a male whose Y chromosome is from an unexposed male. We tested our hypothesis using a mouse model in which F1 generation animals were exposed prenatally to DEHP and the severity of impacted reproductive traits was compared between the F3 generation males that were descendants of F1 males (paternal lineage) and those from F1 females (maternal lineage). In the paternal lineage transmission, the males inherit the Y chromosome from their father only and gene modifications on the Y chromosome will pass from fathers to sons for multiple generations. In contrast, in maternal lineage transmission, the male inherits the X chromosome from the mother and will have an unexposed Y chromosome. Our results show that paternal lineage F3 DEHP males exhibited fertility, testicular steroidogenic capacity, and spermatogenesis outcomes that were more severely impaired than those of maternal lineage males. This transgenerational difference in the DEHP impact can be attributed specifically to the Y chromosome.

## Results

### Prenatal exposure to DEHP affects the body and gonadal weights and testosterone levels of the F3 generation in a lineage- and dose-dependent manner

**Figure 1:**
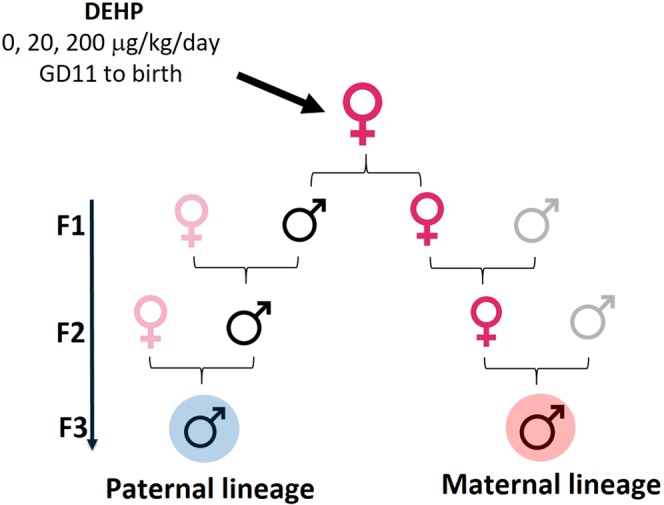
Schematic diagram of the experimental design. The pregnant female mice (F0) were orally dosed with (tocopherol-stripped corn oil (control), or 20 µg/kg/day or 200 µg/kg/day of DEHP from gestational day (GD) 11 to the day of birth. Adult F1 males/females were mated to produce F3 males from paternal and maternal lineages. To examine DEHP transgenerational transmission through the paternal lineage, seven adult F1 males from different litters were randomly selected and naturally mated with non-treated females to generate F2 males for the paternal lines. When the F2 generation males were three months old, seven males from different litters were mated with non-treated females to create the F3 generation from the paternal lineage. By the same pattern, to examine the DEHP transgenerational transmission through the maternal lineage, seven adult F1 females were mated with non-treated males to generate F2 males from the maternal lineage. When the F2 generation females were three months old, seven females from different litters were randomly selected and mated with non-treated males to create the F3 generation males from the maternal lineage.

Pregnant female mice (F0) were orally dosed from gestational day (GD) 11 to the day of birth with either the vehicle control (tocopherol-stripped corn oil), 20 µg/kg/day, or 200 µg/kg/day of DEHP. We chose to dose between GD 11 until birth because this is a critical time for both gonadal development and establishing the germline epigenome^50,51^. Therefore, any alterations caused by the exposure to DEHP may impact the gonadal function of the F1 and future generations. To produce the paternal male line, young adult F1 males were mated with non-treated females to generate F2, and the resulting young adult F2 males were bred with non-treated females to generate F3 generation males. Similarly, maternal lineage F3 males were produced by breeding F1 females with non-treated males and the resulting F2 females were then bred with non-treated males to generate F3 males **(**Fig. 1**)**. The paternal and maternal F3 generation males were used to assess the transgenerational impact of DEHP exposure.

**Figure 2:**
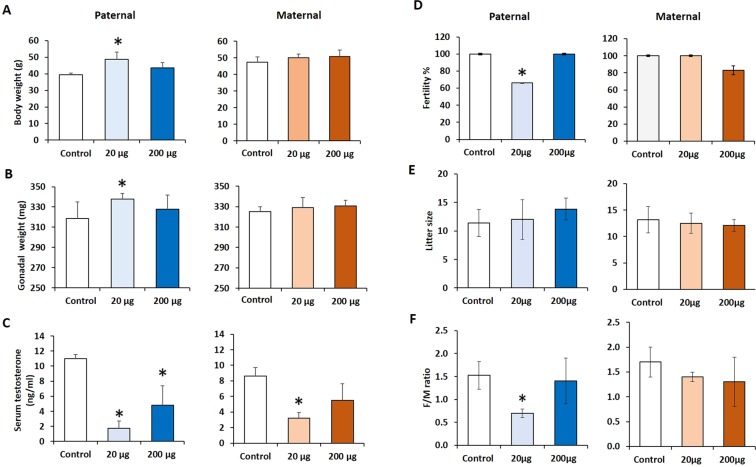
The effects of prenatal DEHP exposure on the body and gonadal weights, serum testosterone level, and fertility of Maternal and Paternal F3 males. (**A**) Body weight (g), **(B)** gonadal weights (mg), **(C)** serum testosterone concentration (ng/ml) were measured. **(D)** Fertility % (percent of males that produced a litter at each trial), **(E)** litter size (numbers of pups per litter), and **[F]** sex ratio (number of females to male pups produced in each litter) were measured. Graphs show mean ± SEM. Asterisks indicate *P* ≤ 0.05 when compared with control group, n = 5 to 7 males/treatment (Control= 7 males; 20 µg/kg/day DEHP group = 5 males; 200 µg/kg/day DEHP group = 5 males).

Paternal lineage F3 male mice from the 20 µg/kg/day DEHP treatment group had heavier body and gonadal weights than the controls (*P* = 0.04, *P* = 0.03; respectively) **(**Fig. 2A,B, n = 5 to 7 males/treatment**)**. In contrast, no significant differences in body and gonadal weights were seen between control and maternal lineage F3 DEHP males. The 200 µg/kg/day DEHP treatment group did not show an alteration in their gonadal and body weights in males from either lineage. These results show that prenatal exposure to DEHP impacts gonadal and body weights in a lineage- and dose-dependent manner. Serum testosterone levels of paternal lineage F3 DEHP males of the 20 μg/kg/day and 200 μg/kg/day DEHP were significantly lower (*P* = 0.01, *P* = 0.05; respectively) compared to the controls **(**Fig. 2C**)**. A similar trend was seen in the maternal lineage F3 males, even though the difference did not reach statistical significance in the 200 μg/kg/day DEHP lineage males **(**Fig. 2C**)**.

### Prenatal exposure to DEHP decreases the fertility of F3 generation males in a lineage- and dose-dependent manner

The transgenerational effects of DEHP exposure on overall gonadal function were assessed by fertility tests. To assess fertility, three-month-old proven breeder female CD-1 mice were purchased from Jackson Laboratory (Bar Harbor, MA) and given a week-long acclimation period. At the age of six months, F3 DEHP males of maternal and paternal lineages were housed with proven breeder females for two weeks and their fertility-related indices were measured **(**Fig. 2D, n = 5 to 7 males/treatment**)**. Paternal lineage F3 DEHP males that were from F1 males prenatally exposed to 20 µg/kg/day DEHP showed lower fertility compared to the controls (*P* = 0.03). However, the fertility of the maternal lineage F3 DEHP males was not different from those of the controls **(**Fig. 2D**)**. The litter size of both the paternal and maternal lineage F3 DEHP male groups was not different from that of the control **(**Fig. 2E**)**. The paternal lineage F3 males from the 20 µg/kg/day DEHP group had a significantly lower female-to-male ratio compared to the control group (P = 0.04) **(**Fig. 2F**)**.

### Prenatal exposure to DEHP decreases the steroidogenic capacity of F3 generation males in a lineage- and dose-dependent manner

**Figure 3:**
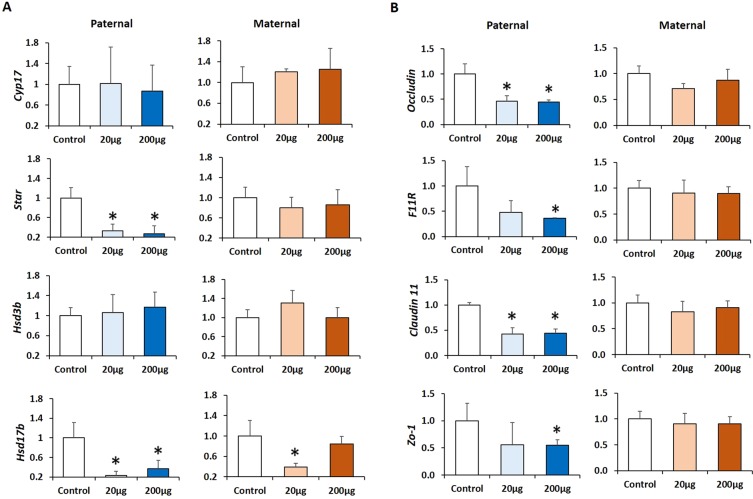
Impact of prenatal exposure to DEHP on steroidogenic and testicular blood testes barriers (BTB) genes expression of Maternal and Paternal F3 males. (**A**) Real time-PCR analysis of testicular steroidogenesis gene (mRNA) expressions in F3 males–paternal and maternal lineages. **(B)** Real time-PCR analysis of testicular blood testes barriers gene (mRNA) expressions (*Occludin, F11R, Claudin 11, and Zo-1*) in paternal and maternal lineage F3 males. Data from each gene were normalized to the corresponding value of the internal control (*L19*), gene expression data are presented as fold changes of each group compared to control. Graphs show mean ± SEM, asterisks indicate *P* ≤ 0.05 when compared with control group; n = 5 to 7 males per treatment group.

The lower testosterone level in the serum prompted us to determine if the testosterone synthesis pathway was transgenerationally affected by the DEHP exposure in a lineage-dependent manner. As the serum testosterone level is predominantly regulated by testicular testosterone synthesis^52^, the expression patterns of the genes that are involved in testosterone synthesis were measured by quantitative PCR. The paternal lineage F3 males of the 20 µg/kg/day and 200 μg/kg/day DEHP treatment groups had significantly lower *Star* and *Hsd17β*1 expression levels than the controls [20 µg/kg/day DEHP group (*P* = 0.01, *P* = 0.008), and 200 µg/kg/day DEHP group *(P* = 0.02, *P* = 0.05), respectively] **(**Fig. 3A**)**. In the maternal lineage F3 males, only those from the 20 µg/kg/day DEHP dose group showed a significantly lower *Hsd17β*1 expression (*P* = 0.04) compared to the control group **(**Fig. 3A**)**. The expression of *Cyp17a*1 and *Hsd3b*1 was not altered in either group of F3 lineage males.

### Prenatal exposure to DEHP alters blood testes barriers gene expression in the F3 generation males

In the testes, the blood testes barrier (BTB) is created by adjacent Sertoli cells that prevent diffusion of any harmful substances to the inside of the testes^53,54^. The BTB also plays a crucial role in spermatogenesis and differentiation of spermatogonia into spermatocytes^53^. The expression of BTB tight junction components (*claudin* 1*1, occludin, Zo-1*, and *F11R*) were assessed by quantitative PCR **(**Fig. 3B, n = 5 to 7 males/treatment**)**. Paternal lineage F3 males from the 200 μg/kg/day DEHP dose group had a significantly lower expression of *claudin 11, occludin, Zo-1*, and *F11R* compared to the control group (*P* = 0.05, *P* = 0.03, *P* = 0.05, *P* = 0.02, respectively). Moreover, the 20 μg/kg/day DEHP treatment group had significantly lower *occludin* and *claudin 11* mRNA expression compared to controls (*P* = 0.01, *P* = 0.008, respectively) **(**Fig. 3B**)**. In contrast, in the maternal lineage F3 males, only the 20 μg/kg/day DEHP group had a lower *occludin* expression (*P* = 0.0 (5compared to control group **(**Fig. 3B**)**. The mRNA expression of *claudin 11, Zo-1*, and *F11R* was not altered in the maternal lineage F3 DEHP males. Utilizing immunohistochemistry, we examined SOX9 expression (Sertoli cell marker) in the testes because Sertoli cells constitute BTB in the seminiferous tubules (Fig. S1). The testes of the paternal F3 male showed decreased number of SOX9-positive cells (Sertoli cells) and the distribution was disorganized compared to control. Expression of SOX9 was reduced in the testes of paternal F3 males compared to control testes.

### Prenatal exposure to DEHP decreased the spermatogenesis of the F3 generation in a lineage-dependent manner

**Figure 4:**
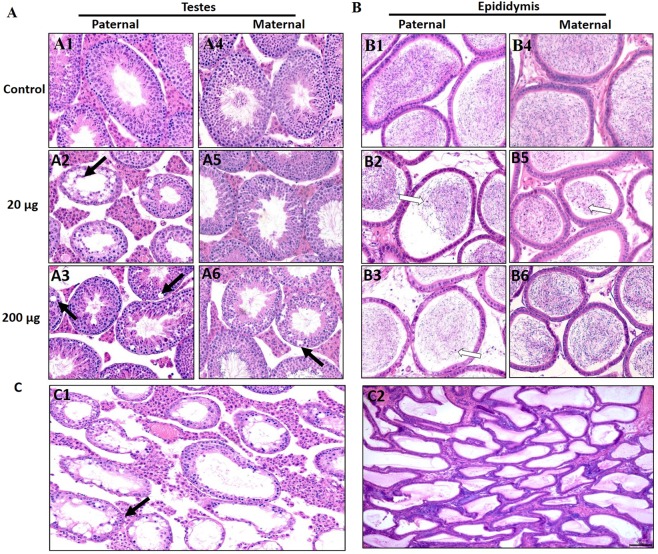
Effect of DEHP exposure on the testes and epididymis of Maternal and Paternal F3 males. (**A**) Testes and **(B)** epididymis was collected at 15 months of age and the epididymis were stained with hematoxylin and eosin, n = 4 to 5 males/treatment. **(**A1,A4**)** Testis of a control mouse. **(**A2,3,5,6) Testes of DEHP treated mice. (B1,B4) Epididymis of a control mouse. **(**B2,3,5,6) Epididymis of DEHP treated mice. **(C)** One mouse from the 20 µg/kg/day DEHP F3 males of paternal lineage had testicular atrophy and sperm stasis with complete absence of any sperm production in the epididymis. Note hypospermatogenesis with degenerative changes in the seminiferous tubules and germ cell degeneration (black arrows), desquamated germ cell the in lumen of epididymis (white arrows).

The impact of prenatal exposure to DEHP on the testes and epididymides of F3 males was microscopically examined. The seminiferous tubules of the controls showed active spermatogenesis **(**Fig. 4A1, n = 4 to 5 males/treatment**)**, and the epididymis contained dense sperm populations **(**Fig. 4B1**)**. However, the testes of the F3 paternal lineage of the 20 µg/kg/day and 200 μg/kg/day DEHP treatment groups exhibited impaired spermatogenesis and degenerative seminiferous tubules **(**Fig. 4A2,A3**)**. Maternal lineage F3 DEHP males also showed degenerative changes on the testes, but to a lesser degree than the paternal F3 males **(**Fig. 4A5,A6**)**. In the epididymides of both paternal and maternal lineage DEHP males, sloughed germ cells were seen in the lumen **(**Figs. 4B, S2). Of note, one mouse from the 20 µg/kg/day DEHP F3 males of paternal lineage had testicular atrophy, spermatocele, and sperm stasis with complete absence of sperm in the epididymis **(**Fig. 4C). Quantitative histological analysis revealed that the paternal lineage F3 DEHP males had a higher number of pathological abnormalities than maternal lineage males **(**Table 1**)**.

**Table 1:** Histopathological impact of transgenerational prenatal DEHP exposure.

	Control	F3- Paternal line		F3- Maternal line	
		20 µg/kg/day DEHP	200 µg/kg/day DEHP	20 µg/kg/day DEHP	200 µg/kg/day DEHP
**Testis**					
-Hypospermatogenesis	0% (0/4)	75% (3/4)	50% (2/4)	20% (1/5)	20% (1/5)
-Germ cell degeneration	0% (0/4)	75% (3/4)	50% (2/4)	20% (1/5)	40% (2/5)
-Abnormal residual bodies	0% (0/4)	75% (3/4)	25% (1/4)	20% (1/5)	20% (1/5)
-Spermatocele	0% (0/4)	25% (1/4)	0% (0/4)	0% (0/5)	0% (0/5)
**Epididymis**					
-Epididymal vacuoles.	0% (0/4)	75% (3/4)	50% (2/4)	20% (1/5)	20% (1/5)
-Germ cell in lumen of epididymis	25% (1/4)	75% (3/4)	75% (3/4)	20% (1/5)	40% (2/5)
-Sperm stasis	0% (0/4)	25% (1/4)	0% (0/4)	0% (0/5)	0% (0/5)

a, the number of mice showing each abnormality per treatment group was divided by the total mice per treatment group to calculate a percentage of affected mice for each abnormality (affected mice/total number of mice).

### Prenatal exposure to DEHP decreased the sperm quantity and quality of the F3 generation in a lineage- and dose-dependent manner

**Figure 5:**
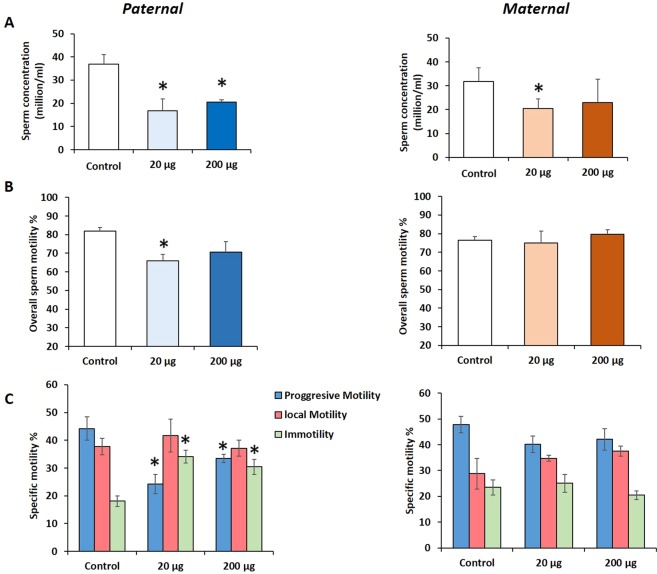
The effects of prenatal DEHP exposure on sperm parameters of Maternal and Paternal F3 males. (**A**) Sperm concentrations (millions/mL), **(B)** sperm motility % (percent of motile sperm), **(C)** different pattern of motility % (progressive motility, local motility and immotile %) were measured. Graphs show mean ± SEM. Asterisks indicate *P* ≤ 0.05 when compared with control group, n = 5 to 7 males/treatment group.

Epididymal sperm concentration and sperm motility were assessed by CASA at 15 months of age. Sperm concentration was significantly decreased in the paternal lineage F3 males of the 20 μg/kg/day and 200 μg/kg/day DEHP groups (*P* = 0.001, *P* = 0.005; respectively) compared to the control **(**Fig. 5A, n = 5 to 7 males/treatment**)**. The maternal lineage F3 males of 20 μg/kg/day DEHP group had also a lower sperm concentration, but to a lesser degree than paternal lineage males (*P* = 0.03). Interestingly, in the paternal lineage F3 males, exposure to 20 μg/kg/day of DEHP led to significantly lower percentages of motile sperm (*P* = 0.03), but no such decreased motility was seen in maternal lineage males **(**Fig. 5B**)**. The percentage of progressively motile sperm was decreased in the 20 μg/kg/day and 200 μg/kg/day DEHP groups (*P* = 0.01, *P* = 0.05; respectively), and increased numbers of immotile sperm were seen in the 20 µg/kg/day, 200 µg/kg/day groups (*P* = 0.03, *P* = 0.04, respectively) in the paternal lineage F3 males **(**Fig. 5C**)**. However, no differences were observed in the sperm motility parameters in the maternal lineage F3 DEHP males **(**Fig. 5B,C**)**.

### Prenatal exposure to DEHP altered testicular gene expression of the F3 generation in a lineage-dependent manner

Testes collected at 15 months of age from control and 20 µg/kg/day DEHP groups (n = 3 males/treatment) from the maternal and paternal F3 males were subjected to RNA sequencing. We chose the 20 µg/kg/day DEHP group as it is the dose that is relevant to daily human exposure to DEHP^15^. The RNA sequencing result showed that a total of 21,353 genes were expressed in the testes. Among them, 320 genes were down-regulated and 122 genes were up-regulated in the paternal F3 DEHP males. Interestingly, of the paternal lineage F3 males, the top 100 most altered genes were all down-regulated as shown in Table 2. The dynein light chain Tctex-type 1A gene (*Dynlt1a*) gene expression was most impacted as determined by fold changes in the paternal lineage F3 males. *Dynt1a* gene is also known as *Tctex-1* (t-complex-associated-testis-expressed 1-like 1) and known to play a role in male germ cell development^55^. In the testes of maternal lineage F3 DEHP males, 77 genes were up-regulated and 23 genes down-regulated **(**Table 3**)**.

**Table 2:** Top 100 differentially expressed genes (DEG) in the testes of DEHP F3 paternal males compared to control group.

Symbol	Description	Fold Change (FC)	P Value
Dynlt1a	dynein light chain Tctex-type 1A	−32.171	3.6E-08
Tmprss11a	transmembrane protease, serine 11a	−14.448	1.5E-02
Klk1b27	kallikrein 1-related peptidase b27	−14.146	1.1E-01
Gm19248	thymosin, beta 10 pseudogene	−10.953	2.6E-01
Klk1b22	kallikrein 1-related peptidase b22	−10.393	1.0E-01
Klk1b24	kallikrein 1-related peptidase b24	−9.919	1.2E-01
Gm6166	fatty acid-binding protein, epidermal-like	−9.901	2.4E-02
Klk1b21	kallikrein 1-related peptidase b21	−9.662	1.2E-01
Gm8220	predicted gene 8220	−7.603	1.2E-04
BC061237	cDNA sequence BC061237	−6.442	1.5E-01
Gm5693	predicted gene 5693	−6.360	8.8E-02
LOC102639037	disks large homolog 5-like	−6.098	1.1E-05
Gm8256	predicted gene 8256	−5.978	2.8E-04
Cd177	CD177 antigen	−5.816	2.1E-05
Zfp33b	zinc finger protein 33B	−5.184	7.5E-07
Gt(pU21)140Imeg	gene trap 140	−4.918	1.4E-01
Gm33677	predicted gene, 33677	−4.650	7.8E-02
Cpa3	carboxypeptidase A3, mast cell	−4.641	4.5E-05
Rtkn2	rhotekin 2	−4.359	9.1E-02
Fam131c	family with sequence similarity 131, member C	−3.669	3.2E-02
Speg	SPEG complex locus	−3.628	3.4E-02
4930579D09Rik	RIKEN cDNA 4930579D09 gene	−3.498	1.7E-01
Klk1	kallikrein 1	−3.487	1.4E-01
1700001G01Rik	RIKEN cDNA 1700001G01 gene	−3.347	1.6E-01
Rps3a3	ribosomal protein S3A3	−3.331	4.3E-01
Aqp2	aquaporin 2	−3.116	5.5E-03
Pcdh9	protocadherin 9	−3.094	1.4E-01
Crisp1	cysteine-rich secretory protein 1	−2.990	4.3E-01
Pop4	processing of precursor 4	−2.857	3.3E-07
Zfp811	zinc finger protein 811	−2.817	1.7E-02
Atp1a3	ATPase, Na+/K+ transporting, alpha 3 polypeptide	−2.775	1.4E-03
Spock1	sparc/osteonectin, cwcv	−2.757	6.0E-02
Cntnap5c	contactin associated protein-like 5C	−2.717	2.7E-01
1700097N02Rik	RIKEN cDNA 1700097N02 gene	−2.692	2.0E-01
Dapp1	adaptor for phosphotyrosine and phosphoinositides	−2.663	4.0E-01
Nipal1	NIPA-like domain containing 1	−2.650	2.8E-02
Gm33433	predicted gene, 33433	−2.640	3.4E-02
Zfp354b	zinc finger protein 354B	−2.636	3.9E-03
Cpne7	copine VII	−2.626	1.4E-01
Gm29779	predicted gene, 29779	−2.595	3.3E-01
Klk1b16	kallikrein 1-related peptidase b16	−2.584	8.6E-02
Myh7	myosin, heavy polypeptide 7, cardiac muscle, beta	−2.575	5.0E-02
Fcgr3	Fc receptor, IgG, low affinity III	−2.570	1.8E-01
Dnah8	dynein, axonemal, heavy chain 8	−2.563	1.2E-04
Gm35110	predicted gene, 35110	−2.561	2.2E-01
Gm32070	predicted gene, 32070	−2.553	1.1E-03
1700049E15Rik	RIKEN cDNA 1700049E15 gene	−2.548	4.9E-01
Lhfpl3	lipoma HMGIC fusion partner-like 3	−2.547	3.9E-01

**Table 3:** Top 100 differentially expressed genes (DEG) in the testes of DEHP F3 maternal males compared to control group.

Symbol	Discription	Fold change (FC)	P Value
1700061I17Rik	RIKEN cDNA 1700061I17 gene	−5.393	2.60E-06
4930503E14Rik	RIKEN cDNA 4930503E14 gene	−11.313	3.40E-06
4933422 A05Rik	RIKEN cDNA 4933422A05 gene	−3.914	6.90E-06
Klk1b21	kallikrein 1-related peptidase b21	2.548	1.20E-05
Mrs2	MRS2 magnesium transporter	1.855	1.70E-05
4930401O12Rik	RIKEN cDNA 4930401O12 gene	−1.892	1.90E-05
1700120G07Rik	RIKEN cDNA 1700120G07 gene	−3.228	2.40E-05
Itih5	inter-alpha (globulin) inhibitor H5	2.028	5.40E-05
Klk1b27	kallikrein 1-related peptidase b27	2.243	5.20E-05
Unc45b	unc-45 myosin chaperone B	3.626	5.50E-05
Klk1b24	kallikrein 1-related peptidase b24	2.166	6.20E-05
4930579D09Rik	RIKEN cDNA 4930579D09 gene	3.606	0.00008
Klk1b22	kallikrein 1-related peptidase b22	3.220	0.00010
Lrfn3	leucine rich repeat and fibronectin type III domain 3	4.593	0.00024
Dera	deoxyribose-phosphate aldolase (putative)	−1.809	0.00042
Bdh1	3-hydroxybutyrate dehydrogenase, type 1	−1.520	0.00047
Tfb1m	transcription factor B1, mitochondrial	1.672	0.00047
Tdgf1	teratocarcinoma-derived growth factor 1	−5.699	0.00068
Slfn5os	schlafen 5, opposite strand	−2.194	0.00070
Kpna2-ps	Kpna2 retrotransposed pseudogene	−4.785	0.00069
Palmd	palmdelphin	−2.074	0.00076
Serpina5	serine (or cysteine) peptidase inhibitor, clade A, member 5	−1.455	0.00090
Gtf2ird2	GTF2I repeat domain containing 2	−1.756	0.00110
Ccl24	chemokine (C-C motif) ligand 24	−2.100	0.00110
Adamts19	a disintegrin-like and metallopeptidase	3.660	0.0011
Rpl35a	ribosomal protein L35A	3.878	0.0012
Lhcgr	luteinizing hormone/choriogonadotropin receptor	−1.333	0.0013
4930548J01Rik	RIKEN cDNA 4930548J01 gene	1.885	0.0013
Pvt1	plasmacytoma variant translocation 1	−1.502	0.0014
Tomm6	translocase of outer mitochondrial membrane 6	−1.395	0.0014
Fmo1	flavin containing monooxygenase 1	2.590	0.0015
A930005H10Rik	RIKEN cDNA A930005H10 gene	−1.608	0.0017
Kcnab2	potassium voltage-gated channel, beta member 2	1.689	0.0019
Esx1	extraembryonic, spermatogenesis, homeobox 1	1.639	0.0019
Sgsh	N-sulfoglucosamine sulfohydrolase (sulfamidase)	1.605	0.0020
Lrg1	leucine-rich alpha-2-glycoprotein 1	−1.485	0.0021
Obp2a	odorant binding protein 2A	−2.554	0.0023
Spink4	serine peptidase inhibitor, Kazal type 4	−1.627	0.0023
Specc1	sperm antigen with calponin homology coil domains 1	−1.366	0.0024
Lifr	leukemia inhibitory factor receptor	1.464	0.0024
Dhcr24	24-dehydrocholesterol reductase	−1.287	0.0025
Zfp951	zinc finger protein 951	−2.112	0.0025
Ifnk	interferon kappa	−3.712	0.0026
Loxl2	lysyl oxidase-like 2	−1.736	0.0027
Tnni1	troponin I, skeletal, slow 1	−1.857	0.0031

### Prenatal exposure to DEHP altered testicular cAMP signaling pathway of the paternal lineage F3 generation

**Figure 6:**
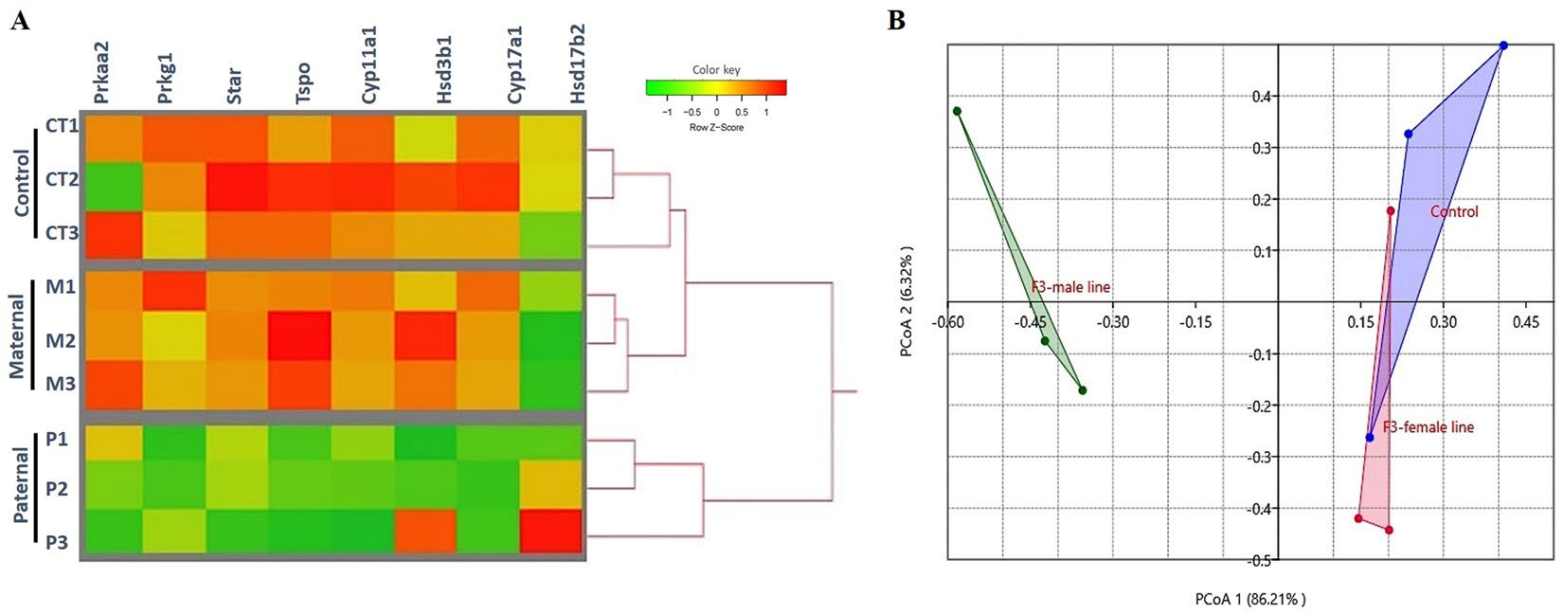
DEHP disrupts cAMP signaling pathway in the F3 paternal lineage males but not in the F3 maternal lineage males. (**A**) Clustering analysis heat map showing log fold change of the cAMP signaling pathway-expressed genes of DEHP F3 maternal and paternal males relative to average control expression. Each row represents a sample, and each column represents a gene. **(B)** Principal coordinate analysis (PCoA) of the differentially expressed genes in the cAMP pathway, the percent of variation explained by each principal coordinate is indicated on the axes. The points represent individual mouse data from each group (n = 3 males/treatment) as: control (blue), F3 DEHP maternal group (red), and F3 DEHP paternal group (green).

Steroid hormone biosynthesis in Leydig cells is regulated through hormone activation of Cyclic AMP (cAMP) signaling pathways^56^. The decreased testosterone level and steroidogenic gene expression prompted us to examine if the cAMP signaling pathway was transgenerationally affected by the prenatal exposure to the DEHP in a lineage-dependent manner. Pathway analysis using the RNA sequencing data revealed a significant down-regulation of mRNAs for protein kinase type I (*Prkg1*), translocator protein (*Tspo*), cytochrome P450, 11a1 (*Cyp11a1*), cytochrome P450, 17a1 (*Cyp17a1*), and hydroxy-delta-5-steroid dehydrogenase 3 beta1 (*Hsd3b1*) of the paternal lineage DEHP F3 males compared to the control, but no such difference was seen in the maternal lineage males **(**Fig. 6A**)**. The expression levels of *Prkg1* and *Tspo* were down-regulated in the paternal lineage F3 DEHP males compared to the controls. Importantly, *Tspo* is involved in regulating cholesterol transport across the mitochondrial membranes^57^. Furthermore, *Prkg1* in the Leydig cells plays an important role in phosphoprotein and activation of *Star* initiated testicular steroidogenesis^58,59^. Principal coordinate analysis (PCoA) showed a variation of 86.2% between controls and DEHP F3 paternal lineage, but only 6.32% variation between control and DEHP F3 maternal lineage **(**Fig. 6B**)**, indicating that cAMP signaling pathway was heavily impacted in paternal lineage, but not in maternal lineage testis. Our results indicate that prenatal DEHP exposure transgenerationally impacts *Tspo* and *Prkg1* expression in Leydig cells, which may inhibit testosterone synthesis in paternal lineage F3 DEHP males.

### Effect of prenatal DEHP exposure on the expression of Y- and X-chromosome genes in the F3 generation

**Figure 7:**
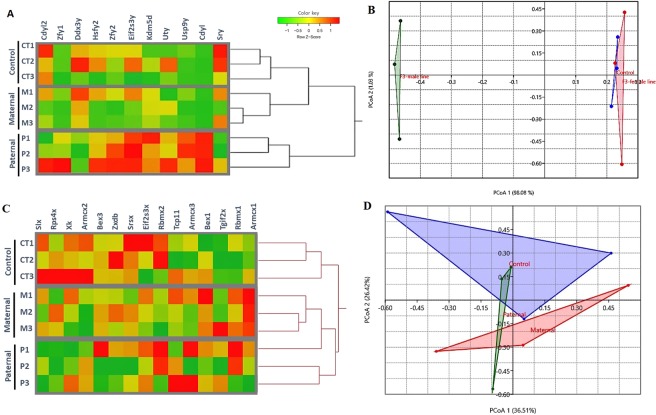
The effects of prenatal DEHP exposure on Y-and X-chromosome genes of Maternal and Paternal F3 males. (**A**) Clustering analysis heat map showing log fold change of Y-chromosome genes of DEHP F3 maternal and paternal males relative to average control expression. Each row represents a sample, and each column represents a gene. **(B)** Principal coordinate analysis (PCoA) of the differentially expressed genes in located in Y chromosome, the percent of variation explained by each principal coordinate is indicated on the axes. **(C)** Clustering analysis heat map showing log fold change of X-chromosome genes of DEHP F3 maternal and paternal males relative to average control expression. **(D)** Principal coordinate analysis (PCoA) of the differentially expressed genes in located in X chromosome, the percent of variation explained by each principal coordinate is indicated on the axes. The points represent individual mouse data from each group (n = 3 males/treatment) as: control (blue), F3 DEHP maternal group (red), and F3 DEHP paternal group (green).

Our results show that ancestral DEHP exposure leads to a transgenerational impact on fertility, testicular steroidogenesis, and BTB integrity in paternal lineage F3 males more than in maternal lineage F3 males. This lineage-dependent transgenerational transmission led us to see if sex chromosome genes were responsible for such differences. The expression patterns of the sex chromosome genes were examined using the testicular RNA-seq data. In the paternal lineage F3 males of the DEHP exposed group, the expression of the sex-determining region of Chr Y gene (*Sry*) was down-regulated, whereas other Y-chromosome genes such as eukaryotic translation initiation factor 2 (*Eif2s3y*), chromodomain protein, Y chromosome-like (*Cdyl*), and Zinc finger protein 2 (*Zfy2*) genes were up-regulated compared to the controls. In contrast, the expression of these genes was not affected in maternal lineage F3 males compared to controls **(**Fig. 7A**)**. PCoA analysis showed a variation of 98.08% on the expression pattern of Y- chromosome genes between the controls and paternal lineage F3 males of DEHP group **(**Fig. 7B**)**, indicating that prenatal exposure to DEHP disrupts Y chromosome genes expression in the paternal lineage F3 males, but not in the maternal lineage. On the contrary, the expression patterns of X- chromosome genes in the paternal lineage F3 males and maternal lineage F3 males of DEHP groups were not different from those of the controls **(**Fig. 7C**)**. PCoA analysis showed that all the three groups clustered together **(**Fig. 7D**)**, confirming that no differences were found in the expression pattern of X-chromosome genes.

## Discussion

It is known that prenatal exposure to DEHP impacts fertility and reproduction of the third (F3) generation^37,38,48,60,61^. Previous studies showed that prenatal exposure to DEHP transgenerationally disrupts testicular germ cell organization and spermatogonial stem cell function in F3 generation males^32,38^. Interestingly, the impacted future generations display a wide range of heterogeneity in their reproductive traits. In this study, the role that Y chromosome plays in creating such heterogeneity was explored by testing the hypothesis that the Y chromosome serves as a carrier of the exposure impact to future generations. Our results show that paternal lineage F3 DEHP males exhibited fertility, testicular steroidogenic capacity, and spermatogenesis outcomes that were more severely impaired than those of maternal lineage F3 males.

In this study, pregnant female mice were orally exposed daily from embryonic day 11 until birth to vehicle control or either 20 μg/kg/day or 200 μg/kg/day of DEHP. The paternal and maternal F3 generation males were used to assess the transgenerational impact of DEHP exposure. Our study showed that paternal lineage F3 male mice from the 20 µg/kg/day DEHP dosing group had body and gonadal weights that were significantly heavier than those of the control mice **(**Fig. 2A,B). In contrast, there were no changes to body or gonadal weight of maternal lineage F3 DEHP males compared to controls, indicating a paternal transmission of the phenotypes to the paternal F3 males. These impacts in the paternal lineage males suggest that the transgenerational effect is carried to next generations via sperm^62,63^. The increased body weight was expected because previous studies reported that both current and future generations that prenatally exposed to DEHP tend to have heavier body weights and develop obesity^38,64^. Furthermore, our previous study showed that prenatal exposure to DEHP increased the gonadal weight of F1 male mice^33^. Indeed, a previous study showed the first evidence that the obesity-resistant phenotypes are transmitted through the paternal lineage, but not the maternal lineage using an obesity-resistant 6C2d congenic strain^63^.

We also observed that paternal lineage F3 males from the 20 μg/kg/day and 200 μg/kg/day DEHP treatment groups had lower serum testosterone levels than the controls. In contrast, in the maternal lineage F3 males, only the 20 µg/kg/day group showed a decrease in testosterone level compared to controls. These findings are in agreement with previous reports documenting the transgenerational effects of DEHP on testosterone production^65^. Previous studies showed that prenatal exposure to DEHP causes androgen deficiency during embryogenesis in both animals and humans^66^, and that the DEHP impact on testosterone production mainly results from excessive production of reactive oxygen species (ROS), contributing to Leydig cell dysfunction^66–68^.

Fertility of the F3 generation males was tested to determine the ultimate consequence of ancestral DEHP exposure on reproductive function. Paternal lineage F3 males in the 20 µg/kg/day DEHP group showed lower fertility compared to controls, whereas no changes were found in the higher dose group (200 µg/kg/day) **(**Fig. 2D**)**. Notably, there was no change in the fertility of maternal lineage F3 DEHP males compared to controls, indicating a lineage-dependent transmission of the phenotype. This result is in line with the findings of our previous study that examined the impact of prenatal exposure to DEHP on the F1 male fertility in mice^33^. The lower fertility in the paternal line may be caused by a problem in sperm motility or sperm DNA fragmentation as a recent study showed that DEHP exposure leads to reduced sperm motility and increased sperm DNA fragmentation^69^. Another possibility is decreased sperm capacitation, a process that is regulated by the cholesterol contents in the sperm membrane^70^. In support, in the paternal lineage F3 DEHP males, *Tspo* expression was downregulated, indicating altered cholesterol contents in the sperm membrane and therefore, sperm capacitation. Taken together, the decreased epididymal sperm motility, the histopathological changes seen in seminiferous tubules, and potentially decreased sperm capacitation may collectively contribute to the lower fertility in the paternal lineage F3 DEHP males.

Previous studies showed that treatment of pregnant females with DEHP resulted in nonlinear, U-shaped, dose-response effects on number of pups and sex ratio in newborn offspring^24,33,71–74^. Our findings indicate that the pattern of nonlinear dose-response seen in the first generation is transmitted to the future generations. Of note, the effects of EDCs are dependent on dose, and importantly, low (physiological) doses can be more effective at altering some endpoints compared with high (toxicological) doses^75^. EDCs, including DEHP, have been shown to exhibit both low-dose and non-monotonic (non-linear) dose effects^75^, possibly by different mechanisms of action at each dose^75^. EDCs mimic endogenous hormones and therefore at low doses, EDCs may act by binding to hormone receptors in a manner similar to that for endogenous ligands^75^. Many well-characterized mechanisms for these dose-specific effects include receptor down-regulation at high doses versus up-regulation at low doses^76^. Although transgenerational exposure to phthalates has been shown to have both low-dose and non-monotonic effects, the mechanism for these effects is still largely understudied^33,34,77–79^.

The low testosterone levels seen along with low fertility in the F3 DEHP males led us to examine whether their machinery for testosterone production was impaired in those males. Testicular steroidogenesis is an important process for synthesizing testosterone, and any dysfunction on this pathway could impact male fertility^52,80–82^. The paternal lineage F3 males in the 20 µg/kg/day and 200 μg/kg/day DEHP groups had significantly lower *Star* and *Hsd17β1* mRNA expression levels than controls. In contrast, in the maternal lineage F3 males, only those from the 20 µg/kg/day DEHP group showed a significantly lower *Hsd17β1* expression compared to controls. Collectively, these results indicate that paternal F3 DEHP males may have higher transgenerational impact on steroidogenic capacity than F3 maternal lineage males. *Star* is responsible for cholesterol transport into the inner mitochondria and its down-regulation is associated with reduced cholesterol uptake, leading to decreased testosterone synthesis^83,84^. Taken together, these results suggest that the low serum testosterone levels seen in the paternal F3 DEHP lineage males may be primarily due to adversely affected testicular steroidogenesis. Our results are consistent with the results of a previous report that observed transgenerational decrease in steroidogenic enzyme expression in DEHP exposed groups^35,36^.

The decreased testosterone level and steroidogenic gene expression prompted us to determine if the cAMP signaling pathway was transgenerationally affected by the DEHP exposure in a lineage-dependent manner. Our results showed that the cAMP signaling pathway was transgenerationally affected by prenatal exposure to the DEHP in a lineage-dependent manner. The expression levels of *Prkg1* and *Tspo* were down-regulated in paternal lineage F3 DEHP males **(**Fig. 6**)**. Importantly, TSPO is involved mainly in regulating cholesterol transport across the mitochondrial membranes^57^. It has been shown that the levels of the TSPO protein in Leydig cells were decreased in testes of adult mice exposed to DEHP compared to controls^85,86^. Furthermore, *PRKG1* in Leydig cells plays an important role in phosphoprotein and activation of *Star* initiate testicular steroidogenesis^58,59^. Our results indicate that prenatal DEHP exposure transgenerationally impacts *Tspo* and *Prkg1* expression in Leydig cell, which may alter testosterone synthesis in paternal lineage F3 DEHP males. However, *Prkg1* and *Tspo* gene expression in the testes of maternal lineage F3 DEHP males was not different from those of the controls, indicating that ancestral exposure to DEHP disrupts the cAMP signaling pathway in DEHP paternal lineage F3 males, but not in maternal lineage males, showing a lineage dependent transmission of the exposure effect.

We examined the possibility of impaired BTB as a factor contributing to the decreased fertility in the F3 DEHP males. Particularly, we were interested in tight junction proteins because they are the key components of BTB, and any disruption of BTB function or integrity leads to testicular injury and infertility^87^. As a result, the expression levels of *claudin 11, occludin, ZO-1*, and *F11R* were decreased in the paternal lineage F3 DEHP males compared to the controls **(**Fig. 3B**)**. In contrast, in the maternal F3 lineage males, only the 20 μg/kg/day group had a lower *Occludin* expression compared to controls, indicating that DEHP F3 paternal lineage males may have more severe impact in their BTB gene expression than maternal F3 lineage males. Indeed, when we stained testis tissue sections with anti-SOX9 antibody (Sertoli cell marker), fewer SOX9-positive cells were seen in the testes of paternal lineage F3 males compared to control testes, and the distribution of the SOX9-positive cells was disorganized (Fig. S2). Interconnected Sertoli cells constitute BTB in the testis. Therefore, fewer and disorganized Sertoli cells indicate that the BTB might be disrupted in the paternal lineage DEHP males, consequently affecting spermatogenesis. Interestingly, the testes of paternal lineage F3 DEHP males had more pathological abnormalities than those of maternal lineage males **(**Table 1**)**, suggesting a paternal transmission of the phenotypes to the F3 males. These results are consistent with a recent rat study that found that DEHP exposure led to decreased occludin expression compared to control in the F1 generation^88^. Because adequate testosterone levels are required for germ cell attachment in seminiferous tubules^88^, the decreased testosterone levels and impaired BTB might contribute to the germ cell detachment and subsequent germ cell apoptosis as seen in previous studies^32,89,90^. Indeed, paternal lineage F3 DEHP males had a lower number of sperm with progressive motility and higher numbers of immotile sperm compared to controls. However, the sperm motility of maternal lineage F3 males was not different from that of the controls **(**Fig. 5**)**. Furthermore, sperm concentrations were lower in the paternal and maternal F3 DEHP males than in the controls **(**Fig. 5**)**. Testosterone level affects sperm motility^91^, hence it is likely that decreased testosterone caused by ancestral DEHP exposure may be partly responsible for the decreased sperm motility. The decreased epididymal sperm motility and the histopathological changes seen in seminiferous tubules could be a factor contributing to the lower fertility observed in the paternal F3 lineage DEHP males. Collectively, DEHP exposure appears to give a lineage-dependent transgenerational impact on BTB integrity that results in more testicular dysfunction and impaired sperm motility on paternal lineage F3 DEHP males compared to those of maternal lineage.

The impaired fertility in the paternal F3 DEHP males led us to examine if the testicular gene expression was impaired in those males. Testicular *Dynlt1a* gene expression was significantly decreased in the paternal lineage F3 DEHP males compared to controls. Interestingly, *Dynlt1a* gene expression of maternal lineage F3 DEHP males was not different from the controls. *Dynlt1a* gene is present in sperm tails, and it is expressed mainly in testis at 200-fold higher levels than in other adult tissues^92–94^. Furthermore, the *Dynlt1a* gene has been linked with male germ cell development and function in mice, and any defects in *Dynlt1a* expression have been linked to defective spermatogenesis in both mouse and Drosophila^55^. Because germ cell maintenance and function are affected by *Dynlt1a* expression, it is likely that decreased *Dynt1a* resulting from ancestral DEHP exposure may be partly responsible for the decreased sperm motility and fertility in paternal F3 DEHP males. Furthermore, testicular zinc finger protein (*Zfp33b, Zfp811, Zfp354b*) was significantly decreased in the paternal lineage F3 DEHP males compared to controls **(**Table 2**)**. Interestingly, *Zfp* gene expression of maternal lineage F3 DEHP males was not different from the controls. Testicular zinc finger protein is a polypeptide comprising 924 amino acid residues^95^, and its transcript is expressed during spermatogenesis^96^. ZFP genes have been found to participate in various biological processes, including signal transduction, transcriptional regulation, RNA binding and morphogenesis, and stress response^97^. A previous study reported that deficiency of *Zfp* in pachytene spermatocytes resulted in undifferentiated spermatogenic cells and decreased male fertility^97^. Additionally, testicular kallikrein 1-related peptide (*Klk1*) expression was significantly decreased in the maternal and paternal lineage F3 DEHP males compared to controls **(**Tables 2, 3**)**. Kallikrein is a glycoprotein involved in the enzymatic activation of kininogens that play a role in sperm motility by stimulating sperm metabolism^98^. Whether this male reproductive dysfunction is an outcome of the defective gene expression of *Dynlt1* and *Zfp* in the paternal germline is yet to be determined.

Collectively, reproductive phenotyping of F3 males shows that prenatal DEHP exposure impacts male fertility, testicular steroidogenesis, and BTB integrity in future generations, preferentially via paternal lineage over maternal lineage. This paternal lineage-dependent transmission strongly supports our hypothesis that the Y chromosome serves as a carrier of the ancestral exposure impact to the future generations. Indeed, in the paternal lineage F3 DEHP males, the mRNA expression of *Sry* was down-regulated, whereas other Y chromosome genes such as *Eif2s3y*, *Cdyl*, and Z*fy2* genes were up-regulated compared to the controls. In contrast, the expression of these genes was not altered in maternal lineage F3 DEHP males. On the contrary, the expression patterns of X- chromosomal genes in the paternal lineage F3 males and maternal lineage F3 DEHP males were not different from controls. Altered expression of Y-chromosomal genes in the paternal F3 DEHP males is likely associated with the lineage-dependent transgenerational transmission phenotype. Notably, male-specific regions of the Y chromosome have been shown to play a critical role in maintaining the fertility through regulation of spermatogenesis^99^. The *Sry* gene is essential for testis development and differentiation and it is expressed in adult testis and even in ejaculated spermatozoa^100^. The *Cdy1* gene is expressed only in testis and it is involved in hyperacetylation of histones during the maturation of spermatids at the final stage of spermatogenesis^101^. Furthermore, *Zfy2* is required for multiple aspects of spermatogenesis, especially for spermatocyte function^102^. Alteration of Y-chromosomal gene expression has been linked to defective spermatogenesis, which could impact testicular development and function^103^. Indeed, early-life DEHP exposure is known to involve epigenomic reprogramming during gonadal development^104^. Therefore, when DEHP-induced epigenetic changes are introduced during early development, they may permanently alter the epigenome in the germ line (both eggs and sperm), and these changes can be transmitted to subsequent generations^36^.

Collectively, F3 DEHP males from both paternal and maternal lineages had lower testosterone levels and sperm concentrations. However, paternal lineage F3 DEHP males exhibited lower fertility, testicular steroidogenic capacity, and spermatogenesis than those of maternal lineage males. These lineage-independent as well as lineage-dependent transgenerational effects suggest that while autosomes and X-chromosomes may serve as the carriers of the impact of the exposure, the Y-chromosome is a definite carrier of the exposure impact. Future studies should examine if Y chromosomal genes undergo epigenetic changes upon embryonic exposure to DEHP and if so, how that happens.

## Materials and Methods

### Chemicals

DEHP (99% purity) was purchased from Sigma-Aldrich (CAS Number, 117-81-7; St. Lois, USA). Tocopherol-stripped corn oil (the vehicle) was purchased from MP Bio Medicals (Solon, OH). Stock solutions of DEHP were prepared by diluting it in the vehicle to obtain the desired concentrations. The lowest DEHP dose (20 µg/kg/day) was selected because it is the US Environmental Protection Agency (EPA) reference dose for human exposure^15^, and this dose has been previously shown to affect female reproductive parameters^105^. The 200 µg/kg/day was selected because prenatal exposure to these levels has been shown to affect reproduction and induce premature reproductive senescence in male mice^33,65,106^. Further, occupational exposure has been shown to reach these levels^107^.

### Animals and dosing regimen

All animal procedures were conducted in accordance with the NIH Guide for the Care and Use of Laboratory Animals and approved by the Institutional Animal Care and Use Committee of the University of Illinois at Urbana-Champaign (UIUC). Animal handling and procedures were approved by the UIUC Institutional Animal Care and Use Committee (Animal Protocol ID #: 14144). Adult male and female CD-1 mice were purchased from Charles River Laboratories (Wilmington, MA). Mice were acclimated to the UIUC animal care facility for at least two weeks before use under 12-hour light/dark cycles. The mice were provided with Teklad Rodent Diet 8604 (Harlan) and had free access to food and high-purity water (reverse osmosis filtered) *ad libitum*. 21 pregnant female dams (F0) were prepared by mating two-month-old females with proven breeder males. A female was considered pregnant when a vaginal sperm plug was detected, at which point females were separated from males and individually housed. These dams were considered to be the F0 generation. On GD 11, F0 dams were randomly assigned to three different treatment groups (7 F0 dams/treatment group) and then they were dosed every morning at the same time until the dams gave birth to pups. The pregnant female mice (F0) were orally dosed with the vehicle control (tocopherol-stripped corn oil), 20 µg/kg/day, or 200 µg/kg/day of DEHP by placing a pipette tip into the mouth as previously described^105^. We chose to dose between GD 11 and birth because this is a critical time for epigenetic remodeling and gonadal development in mice^50,51^. Therefore, this exposure time provided a vulnerability for the disruption of normal epigenetic signals and the appearance of adverse effects from DEHP exposure. The pups born to the F0 dams were considered the F1 generation. Therefore, the F1 generation was exposed to DEHP *in utero*.

To examine DEHP transgenerational transmission through the paternal lineage, seven adult F1 males from different litters were randomly selected and naturally mated with non-treated females to generate F2 males for the paternal lines. When the F2 generation males were three months old, seven males from different litters were mated with non-treated females to create the F3 generation from the paternal lineage. By the same pattern, to examine the DEHP transgenerational transmission through the maternal lineage, seven adult F1 females were mated with non-treated males to generate F2 males from the maternal lineage. When the F2 generation females were three months old, seven females from different litters were randomly selected and mated with non-treated males to create the F3 generation males from the maternal lineage **(**Fig. 1**)**. The F3 generations of maternal and paternal lineages were not exposed directly to DEHP. The paternal and maternal F3 generation males were used to assess the transgenerational impact of DEHP exposure. Our previous study showed that prenatal exposure to DEHP accelerates reproductive aging and induces premature reproductive senescence in male mice^33^. We followed the F1 generation males to 22 months old, as no obvious phenotype was seen at younger ages. Therefore, in this study, we kept the F3 males for more than one year so that we could follow their reproductive function at similar time-points as assessed in the F1 generation.

### Body weight and tissue collection

At 15 months of age, F3 mice were euthanized by CO_2_ asphyxiation followed by cervical dislocation, and tissues were collected. Body weight (g) and gonadal (mg) weight were determined. After the mice were euthanized, the testes and epididymis were removed, cleaned, and weighed. One testis was fixed in Bouin’s solution to use for histological evaluation as described below. The other testis was snap-frozen and stored for genomic analysis. Blood was also obtained during collections, and sera were used for hormone assays as described below.

### Measurement of serum testosterone concentration

Peripheral blood was collected at 15 months of age by cardiac puncture. The blood was centrifuged at 2000 × g, and then serum was collected and preserved at −20 °C until further analyses. ELISA kits (DRG Diagnostic) with a reportable range of 0.06–25 ng/ml were used to measure the concentrations of circulating testosterone. The intra- and inter-assay coefficients of variability were less than 10%.

### Fertility test (mating study)

To assess fertility, three-month-old proven breeder female CD-1 mice were purchased from Jackson Laboratory (Bar Harbor, Maine) and given a week-long acclimation period. At six months of age, each F3 male mouse of maternal or paternal lineage was housed with a breeder female for two weeks or until a vaginal sperm plug was observed. The fertility percent (number of males that produce litter/total number of males × 100), litter size (number of pups per litter), and sex ratio (numbers of female/numbers of male pups) were recorded as described in previous studies^108^.

### Steroidogenic gene and blood testes barrier gene expression analysis

Testes were collected at 15 months of age and snap-frozen for quantitative real-time polymerase chain reaction (qPCR) analysis. Total RNA was extracted using TrizolVR solution (Ambion, Carlsbad, CA) and then purified with a RNeasy Kit (Qiagen, Valencia, CA). Concentration and quality of total RNA was analyzed using a Nanodrop (Thermo Scientific, Waltham, MA) and stored at −80 °C until use. Complementary DNA was generated by M-MLV Reverse Transcriptase (Thermo Scientific). PCR reactions were performed with Power SYBR Green PCR Master Mix (Applied Biosystems) according to the manufacturer’s protocol. Fluorescence was measured using the ABI prism 7500 quantitative real-time thermocycler (Applied Biosystems). PCR primers used in this study are presented in Table 4.

**Table 4:** Primer sequences used for RT-PCR.

Gene name	Symbol	Forward primer (5′-3′)	Reverse primer (5′-3′)	Fragment size (bp)
Steroidogenic acute regulatory protein	*Star*	CAGGGAGAGGTGGCTATGCA	CCGTGTCTTTTCCAATCCTCTG	262 bp
Cytochrome P450 11A1	*Cyp11a1*	AGATCCCTTCCCCTGGTGACAATG	CGCATGAGAAGAGTATCGACGCATC	192 bp
3β-hydroxysteroid dehydrogenase 1	*Hsd3b1*	CAGGAGAAAGAACTGCAGGAGGTC	GCACACTTGCTTGAACACAGGC	280 bp
17β-hydroxysteroid dehydrogenase 1	*Hsd17b1*	ACTGTGCCAGCAAGTTTGCG	AAGCGGTTCGTGGAGAAGTAG	310 bp
Cytochrome P450 17A1	*Cyp17a1*	CCAGGACCCAAGTGTGTTCT	CCTGATACGAAGCACTTCTCG	250 bp
*Claudin 11*	CLDN11	GCCATCTTGCTGCTGTTGAC	CGGTGGGCACATACAGGAAA	158 bp
Occludin	*OCLN*	TTGAACTGTGGATTGGCAG	CAAGATAAGCGAACCTTGGC	90 bp
Tight Junction Protein *ZO*-*1*	*ZO-1*	GCGGGGTCGGATCGCCTT	AAACCCAGGAGCCCTGTGAA	289 bp
*F11 Receptor*	*F11R*	AACTGTAATGGGCACCGAGG	TAGGGAGCTGTGATCTGGCT	252 bp
Ribosomal Protein L19	*Rpl19*	CCTGAAGGTCAAAGG GAAT	GTCTGCCTTCAGCTTGTG GA	73 bp

The mRNA expression levels of steroidogenic acute regulatory protein (*Star*), cytochrome P450 cholesterol side-chain cleavage (*Cyp11a1*), 3β-hydroxysteroid dehydrogenase 1 (*Hsd3b1*), 17β-hydroxysteroid dehydrogenase 1 (*Hsd17b1*), and cytochrome P450 aromatase (*Cyp17a1*) were measured by real-time PCR. The blood-testis barrier gene expressions (*claudin 11, occludin, ZO-1*, and *F11R*)^87^ was also examined. Expression data were generated using the mathematical standard comparative (ΔΔCt) method. Data from each gene were normalized to the corresponding value of ribosomal protein *L19* (*Rpl19*) and used as the internal control to calculate relative fold changes, which were used for statistical analysis. The ΔCt was calculated by subtracting the *L19* Ct value from the Ct value for the gene of interest^109,110^. The ΔΔCt was calculated from the difference between the ΔCt between the treatment groups and the control group. The relative fold-change of expression was then equated to 2(−ΔΔCt) for each group^109,110^.

### Testicular histopathology

The testis and epididymis were collected at 15 months of age, then fixed in Bouins solution (Ricca chemical Co.) for 24 hours, then transferred to 70% ethyl alcohol until tissue processing. The tissues were embedded in paraffin, sectioned at 7 µm thickness, stained with hematoxylin and eosin, and examined using light microscopy (Olympus BX 51)^33^. Of note, one mouse from the 20 µg/kg/day DEHP F3 males of paternal lineage had testicular atrophy, spermatocele, and sperm stasis with complete absence of sperm in the epididymis **(**Fig. 4C**)**. We considered this mouse that showed major disruption in the testes as an outlier and excluded it from further analysis in an effort to avoid confusion. Quantitative analysis of histopathological abnormalities was done by counting these abnormalities in each testis and epididymis from each mouse in all groups and calculating the percent of affected mice for each abnormality (affected litters /total number of litters)^33,78^. The expression of anti-DDX4 (germ cell marker) antibody in the epididymis and anti-SOX9 (Sertoli cell marker) in the testes was determined by immunohistochemistry. Antigen retrieval for IHC was done using citrate buffer (pH 6.0) and microwaved at 10% power for 15 minutes. Endogenous peroxidase activity was blocked using 3% H_2_O_2_ for 20 minutes, slides were blocked with 5% goat serum for 1 hour before incubating with primary antibodies overnight at 4 °C. DDX4/MVH (Rabbit-anti-DDX4/MVH, AB13840 Abcam) or SOX 9 (Rabbit anti-Sox9, Abcam) primary antibodies were used at 1:2000 concentration. Peroxidase conjugated goat anti-rabbit secondary antibodies were used at 1:200 concentration and detected using a DAB kit (VectorLabs).

### Semen analysis

Semen was analyzed at 15 months of age in the F3 generations of paternal and maternal lineages. For semen analysis, the cauda of the left epididymis was excised and minced with fine scissors in a warm (37 °C) phosphate-buffered saline. The sperm suspension was incubated at 37 °C for 10 minutes to allow spermatozoa to swim out of the minced epididymis. Sperm motility was then analyzed by a computer-assisted sperm analyzer (CASA; Sperm Vision II, Minitube of America, Vernon, WI, USA). Sperm suspensions were loaded onto pre-warmed chamber slides (depth, 100 μm) (Leja slide, Spectrum Technologies, USA) and placed on a warmed microscope stage at 37 °C. At least ten microscopic fields, covering the entire viewable area of the semen analysis chamber without overlapping successive fields, were examined^33^. Sperm motility was measured by the percentage of motile sperm, percent of progressive motile sperm, and percentage of immotile sperm^111^.

For total sperm counts, two aliquots of semen samples were collected from each mouse and diluted in 1:200 of formalin for immobilization. Sperm numbers were counted using a hemocytometer and the average number of sperm concentration per milliliter was calculated and reported as million sperm/mL^111^. To determine the degree of morphological abnormalities, wet mount sperm slides were prepared on clean, grease-free slides containing buffered formalin with eosin nigrosine stain. We then examined 100 sperm per sample under an oil immersion lens using a light microscope^112^.

### RNA sequencing analysis

Frozen testes collected at 15 months of age from the control group and the 20 µg/kg/day group (n = 3 testes/treatment group) from the maternal and paternal F3 males were used for RNA sequencing. Total RNA was extracted using TRIzol Reagent (Invitrogen, Carlsbad, CA) and purified through RNeasy columns (Qiagen, Valencia, CA) according to the manufacturer’s directions. The integrity of total RNA was verified by visualizing the intact and distinct 28S and 18S rRNA bands on a 1.5% agarose gel. Concentrations of RNA were measured with a NanoDrop 1000 spectrophotometer (Thermo Scientific, Waltham, MA). RNA sequencing was then performed at the Genomic Services laboratory of the Roy J. Carver Biotechnology Center at the University of Illinois at Urbana-Champaign.

Raw reads were checked for quality using FASTQC (v 0.11.5), then trimmed and filtered using Trimmomatic (v 0.36) to remove residual adapter content, low-quality bases (Phred quality score <28), and resulting reads shorter than 30 nt. Trimmed/filtered reads were aligned to NCBI’s Mus musculus GRCm38.p6 genome and gene model annotation release 106 using STAR (v 2.5.3a). Post-alignment gene counts were then determined for each NCBI EntrezGene ID using feature Counts from Subread (v 1.5.2-pl) with multi-mapping reads excluded. The raw read counts were input into R (v 3.4.3)^113^ for pre-processing and analysis together using Bioconductor packages^114^ as listed below. There were ~25 million reads aligned uniquely within the 41,595 M. musculus genes. The read counts were normalized by log2 counts per million (log-CPM) values followed by the exclusion of very lowly expressed genes with a negative log-CPM value using edgeR package (v 3.20.5)^115^. Differential expression analysis was conducted using the Voom-limma pipeline with empirical Bayes moderation^116^. The resulting P-values were adjusted using the Benjamini-Hochberg method. Results were expressed as the fold change (FC) of the average expression. A gene was identified as a DEG if its log2 (FC) was higher than 1 and false discovery rate (FDR; adjusted p-value) was lower than 0.05. Principle components analysis and clustering of the differential expression genes on different pathway were analyzed by using JMP 13 software (SAS Institute Inc., North Carolina, USA).

### Statistical analysis

The data were analyzed using the statistical software package SPSS version 22. The comparison was between control and treated groups and the same age point, and the statistical sampling unit was the litter. Multiple comparisons between normally distributed continuous experimental groups were analyzed by the one-way analysis of variance (ANOVA) as a parametric test followed by the Dunnett (two-sided) post hoc test. Multiple comparisons between non-normally distributed experimental groups were analyzed by Kruskal-Wallis as a nonparametric test. Fertility data in each treatment group were statistically compared to the control group using Fisher’s exact test for each treatment group against the control group. The number of animals used for statistical analyses ranged between five to seven mice during the entire experimental period. The data are presented as mean ± SEM. Statistical significance was accepted when *P* values were less than or equal to 0.05.

## Supplementary information

Supplementary data.
