# Supplementary material for: Germline-dependent transmission of male reproductive traits induced by an endocrine disruptor, di-2-ethylhexyl phthalate, in future generations
Source: Sci Rep. 2020 Mar 31;10:5705. doi: 10.1038/s41598-020-62584-w (PMC7109079; doi:10.1038/s41598-020-62584-w)
Supplement: Supplementary file 1 — Supplementary data. [file 41598_2020_62584_MOESM1_ESM.docx]

**Germline-dependent transmission of male reproductive traits induced by an endocrine disruptor, di-2-ethylhexyl phthalate, in future generations**

Radwa Barakat^1, 2^, Po-Ching Lin^1^, Chan Jin Park^1^, Mohamed Zeineldin^3^, Sherry Zhou^1^, Saniya Rattan^1^, Emily Brehm^1^, Jodi A. Flaws^1^, and CheMyong J. Ko^1*^

**^1^** Department of Comparative Biosciences, College of Veterinary Medicine, University of Illinois at Urbana-Champaign, IL 61802, USA; **^2^** Department of Toxicology and Forensic Medicine, College of Veterinary Medicine, Benha University, Qalyubia,13518, Egypt. ^3^ Carl R. Woese Institute for Genomic Biology, University of Illinois at Urbana-Champaign, Urbana, IL, 61801, USA.

*To whom correspondence should be addressed at the Department of Comparative Biosciences, University of Illinois at Urbana-Champaign, 2001 S. Lincoln Ave., Urbana, Illinois 61802. Fax: (217) 244-1652. Email: [jayko@illinois.edu](mailto:jayko@illinois.edu)

**Supplementary Figures**


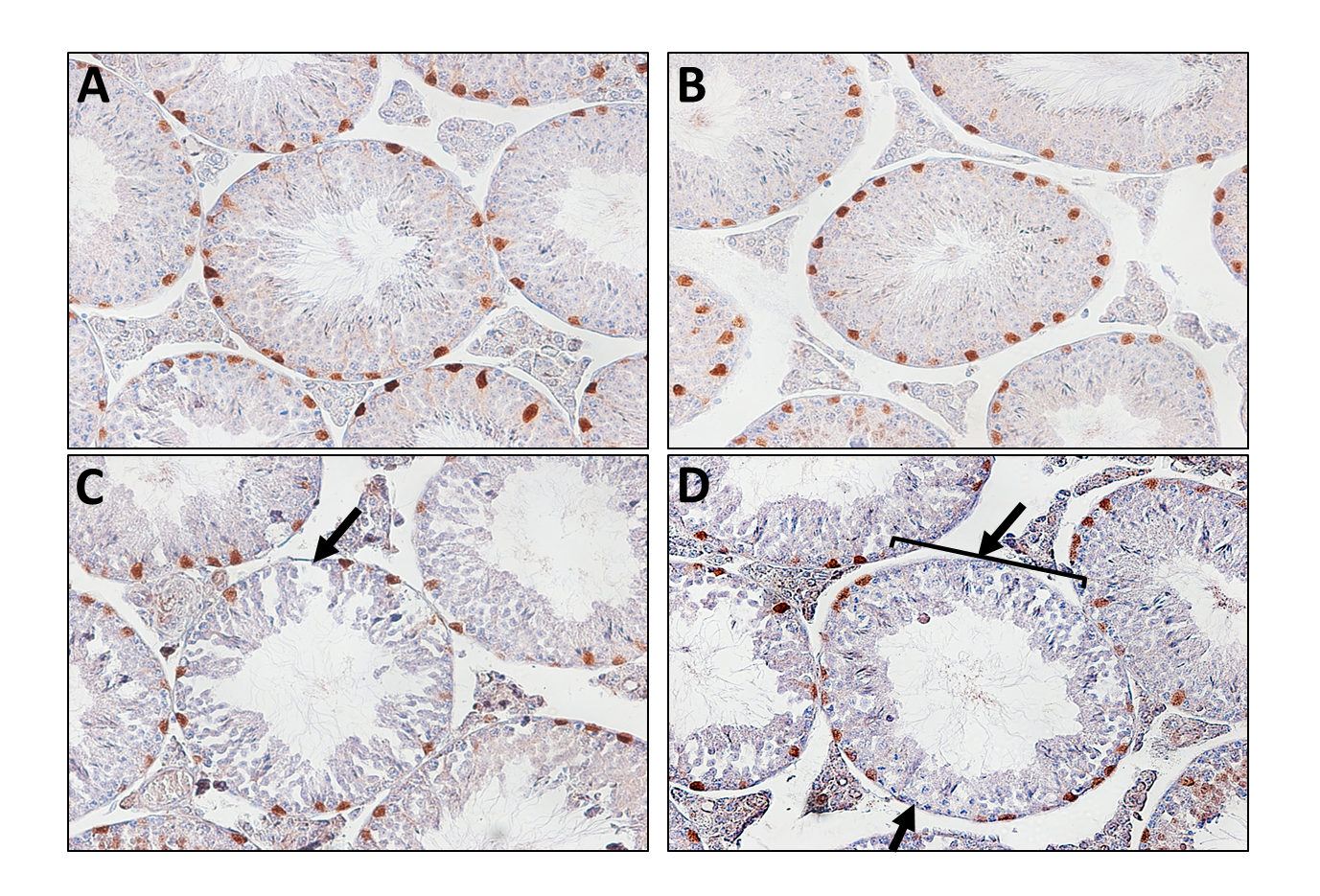


**Supplementary Figure S1. Immunohistochemical detection of SOX9 (Sertoli cell marker) in the testes.** Brown staining represents the site of Sox9 immunostaining in of the testes at 200x magnification. **(A)** Testes of a control mouse. **(B)** Testes of the maternal F3 DEHP males. **(C, D)** Testes of the paternal F3 DEHP males. Black arrows point to reduced expression of SOX9 in the testes of paternal F3 males compared to control.


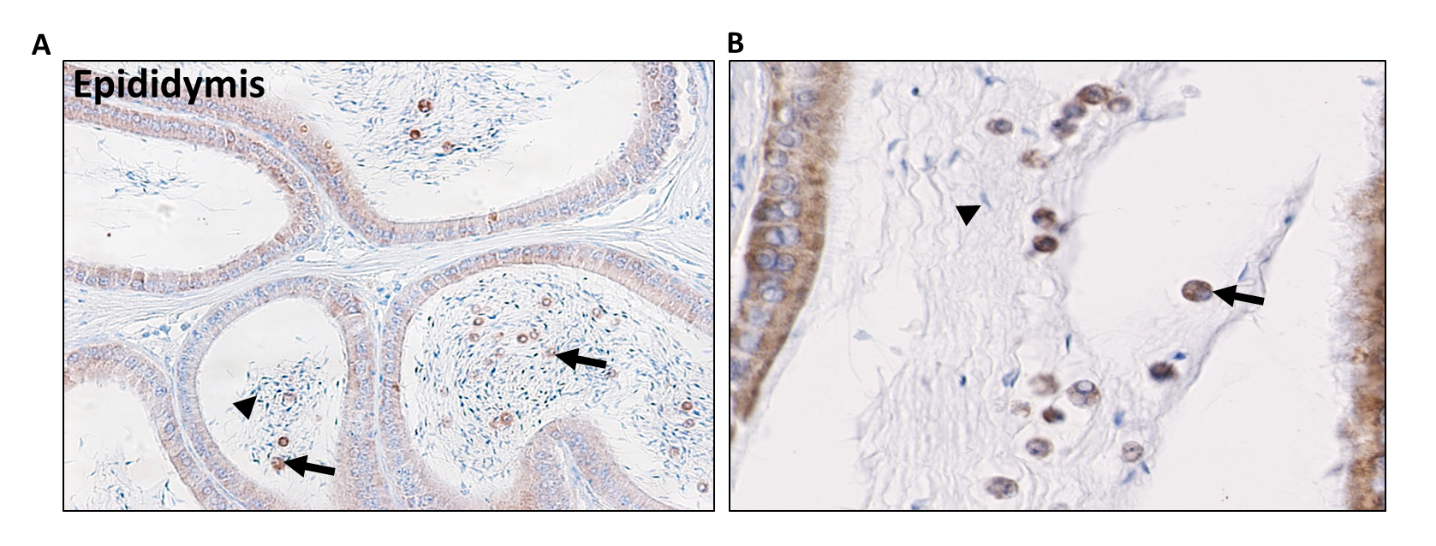


**Supplementary Figure S2. Immunohistochemical detection of DDX4 (germ cell marker) in the epididymis. (A)** Brown staining represents the site of DDX4 immunostaining in of the epididymis of the 20 µg/kg/day DEHP treated groups at 200x magnification. **(B)** Higher magnification of the sloughed gem cells in the lumen at 400X. Black arrows point to sloughed germ cell in the lumen of epididymis. Arrow heads point to normal sperm.
